# Oral Microbe Community and Pyramid Scene Parsing Network-based Periodontitis Risk Prediction

**DOI:** 10.1016/j.identj.2024.10.019

**Published:** 2024-11-28

**Authors:** Zhuo Zhao, Xiaoxu Liu, Mengting Li, Jinjun Liu, Zheng Wang

**Affiliations:** a Key Laboratory of Shaanxi Province for Craniofacial Precision Medicine Research, College of Stomatology, Xi'an Jiaotong University, Xi'an, China; b Clinical Research Center of Shaanxi Province for Dental and Maxillofacial Diseases, College of Stomatology, Xi'an Jiaotong University, Xi'an, China; c Department of Physiology and Pathophysiology, School of Basic Medical Sciences, Xi'an Jiaotong University, Xi'an, China; d State Key Laboratory for Manufacturing System Engineering, Xi'an Jiaotong University, Xi'an, Shaanxi, China

**Keywords:** Periodontitis, Oral microbe, Prediction model, Deep learning

## Abstract

**Background:**

Periodontitis (PD) is a common chronic inflammatory disease affecting the gums and supporting tooth structures. It is often diagnosed only after significant irreversible tissue damage – such as gum recession and bone loss – has occurred, leading to tooth loss and systemic complications. Early detection of PD risk is therefore critical. This study integrates the Pyramid Scene Parsing Network (PSPNet), a deep learning model, with dental plaque microbial profiling data to generate a Periodontitis Risk Score (PRS) for identifying individuals at high risk of developing PD.

**Methods:**

Microbial profiling data from dental plaque samples of 90 healthy controls (CON) and 514 PD patients were obtained from the Gene Expression Omnibus database (GSE32159). A preprocessing algorithm identified predictive indicators for PD and calculated actual PRS values (PRS_Actual_) for both groups. The maximum theoretical PRS was set to ‘1’ for clinically diagnosed PD patients and ‘0’ for CON. The differential algorithm was embedded into PSPNet, which was trained using the generated dataset. The model's predictive ability was evaluated by comparing PSPnet-based PRS (PRS_PSPnet_) with PRS_Actual_.

**Results:**

After preprocessing, 27 indicators were identified for PD risk prediction. The PRS_Actual_ range ranged from 0.011 to 0.524 (mean 0.485) for CON and from 0.589 to 0.700 (mean 0.682) for PD patients, successfully distinguishing between the groups. The mean absolute error between PRS_PSPnet_ and PRS_Actual_ was 0.027, with an average computation time per sample of 10^–5^ seconds, demonstrating both accuracy and efficiency.

**Conclusion:**

By combining microbial profiling with PSPNet, this study offers a reliable, efficient, and noninvasive method for early screening of individuals at high risk of PD. This approach can help prevent irreversible periodontal damage, improve oral health, and reduce the associated health and economic burdens.

## Graphical abstract

**Figure:**
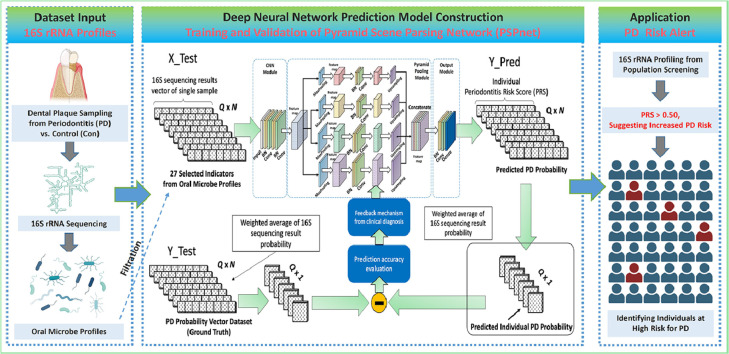

## Introduction

Periodontitis (PD) is a severe chronic inflammatory disease affecting the gums and supporting structures of the teeth. It is the leading cause of tooth loss globally and has significant implications for systemic health.^1^, ^2^, ^3^, ^4^ PD is often diagnosed only after substantial tissue damage has occurred, such as gum recession and bone loss.^5^^,^^6^ Once these irreversible damages develop, they continue to progress, and periodontal treatment can only slow the disease's advancement without restoring lost tissues.^7^ Therefore, early detection and diagnosis of an individual's predisposition to PD are critically important.

An imbalance in the oral microbial community, known as dysbiosis, is closely linked to an increased risk of PD.^8^ Advances in microbial profiling technologies have provided powerful tools for analysing and understanding the unique composition of microbial communities at the individual level,^9^^,^^10^ offering great potential for PD risk stratification. By examining unique microbial profiles in oral cavity, researchers can identify significant differences between healthy individuals and those with PD, enabling personalized oral health guidance. However, traditional medical data processing methods often rely on the abundance of individual bacterial species as predictive biomarkers, limiting their ability to handle large and complex datasets. These methods failed to capture the holistic patterns of the oral microbiome, making it challenging to leverage the comprehensive data from profiling technologies for disease prediction based on the entire microbial community composition.

Artificial intelligence, particularly deep learning – a subset of artificial intelligence – has achieved remarkable success in various domains, including natural language processing, autonomous vehicles, image and video analysis, recommendation systems, robotics, and medical diagnosis.^11^, ^12^, ^13^, ^14^, ^15^ Deep learning models excel at handling complex datasets and extracting features from a holistic perspective.^15^^,^^16^ The Pyramid Scene Parsing Network (PSPNet) is one such model that is highly effective for tasks requiring detailed scene understanding and contextual analysis. PSPNet has been successfully applied in fields such as autonomous driving, medical image analysis, remote sensing, and agriculture.^17^^,^^18^ PSPNet's ability to capture detailed context and handle complex datasets makes it ideal for analysing diverse microbial data. This enables a comprehensive assessment of the oral microbiome for early PD diagnosis, rather than focusing solely on individual bacterial species.

In this study, we employed PSPNet in combination with microbial profiling data to generate a Periodontitis Risk Score (PRS) to help screen and identify individuals at high risk of developing PD. By enabling early detection and diagnosis, our economical, fast, and easily accessible method can alert high-risk individuals to adopt comprehensive oral health management before irreversible periodontal damage occurs. This approach has the potential to drastically reduce the health and economic burdens associated with PD (Graphical abstract).

## Materials and methods

### Ethics approval and consent to participate

As this study involved only the analysis of publicly available data from the Gene Expression Omnibus (GEO) database and did not include direct interaction with human subjects or animal models, it was exempt from ethics approval by the Ethical Committee of Xi'an Jiaotong University.

### Study design

This observational study adhered to the Strengthening the Reporting of Observational Studies in Epidemiology guidelines. The Strengthening the Reporting of Observational Studies in Epidemiology checklist is provided as a Supplemental Table 1.

### Data sources

The data underlying this article are available in the article and in its online Supplemental Material. The abundance matrices of oral microbes in current study were obtained from the GEO database under accession number GSE32159 (https://www.ncbi.nlm.nih.gov/geo/query/acc.cgi?acc=GSE32159).

### Prediction mechanisms and data preprocessing

**Fig. 1:**
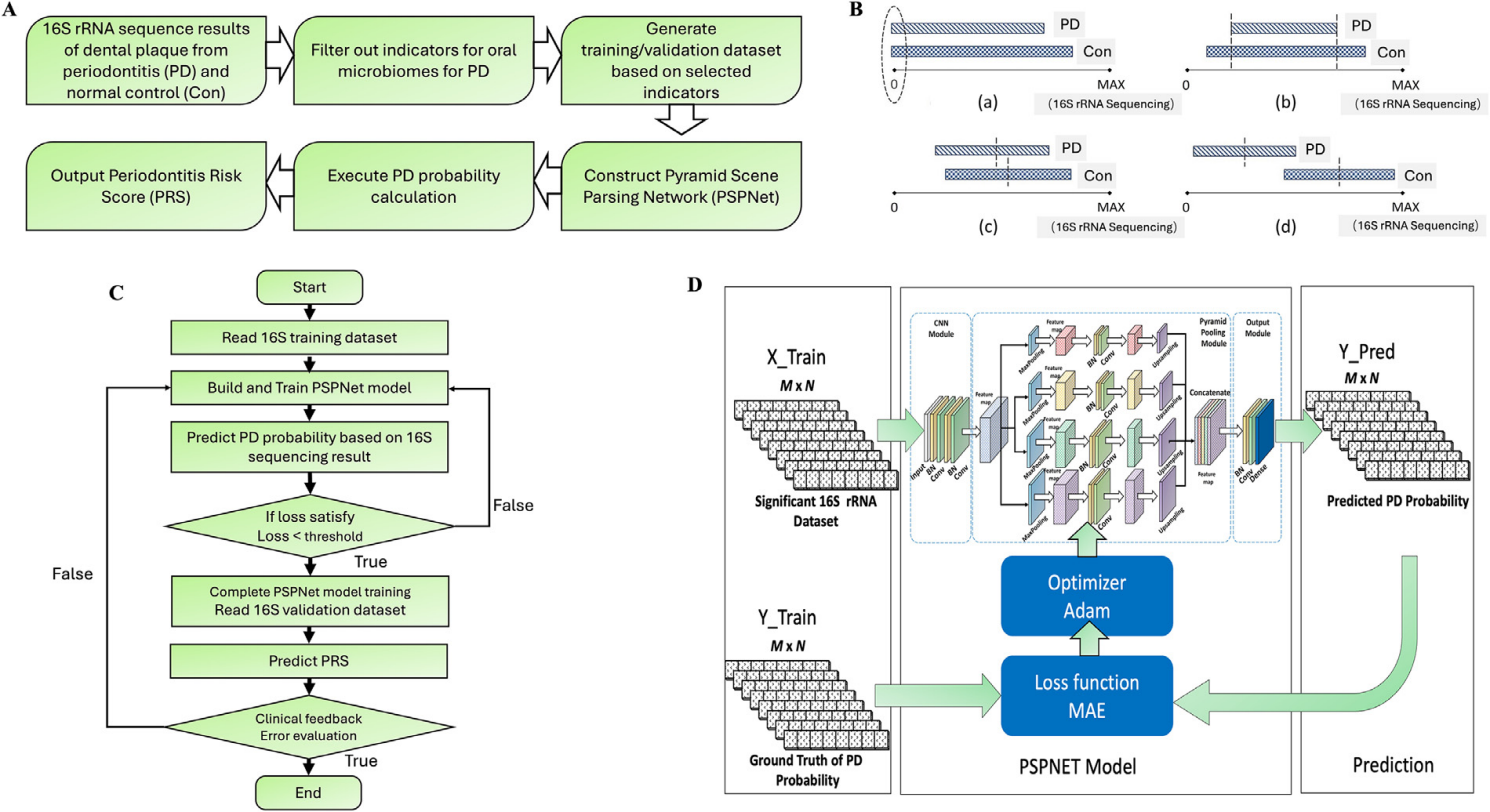
Design and workflow for reporting periodontitis risk score (PRS) via PSPNet. (A) Periodontitis risk score (PRS) prediction methodology; (B) data filtration principles; (C) workflow of PD prediction; (D) the PSPNet architecture, consisting of an input layer, a series of residual convolution blocks, and output layers. PD, periodontitis; Con, normal control; PRS, periodontitis risk score. Components include Conv (2D convolution), Dense (fully connected layer), BN (batch normalization), DP (dropout), MP (max pooling), ConvT (2D deconvolution), and TU (fine feature extraction modules); X_Train: oral microbes abundance matrices for the training dataset; Y_Train: probability vectors corresponding to PD risk; Y_Pred: predicted probability vectors generated by PSPNet; M × N: dataset configuration with M profiles, each containing oral microbes abundance indicators (filtered) of size 1 × *N*.

We formulated PD prediction as a data regression problem by establishing a mapping between biofilm communities and probability vectors. To ensure precise predictions, we initially preprocessed the microbial data to filter out markers showing significant differences between PD and CON. These filtered markers were then used to construct training and validation datasets for our neural network model. The trained network is designed to predict PD risk by generating a Periodontal Risk Score (PRS) based on variations in individual microbial profiles (Figure 1A).

The cleaned and standardized dataset (GSE32159) includes dental plaque samples from 90 CON and 514 PD patients. Our preprocessing steps involved the following (Figure 1B):

1) Removing bacteria with zero expression in both PD and CON groups.
2) Excluding bacteria with completely overlapping expression in both groups.
3) Calculating the mean deviations between expression distributions in the PD and CON groups.
4) Identifying significant differences based on overlapping distribution ratios.

### Dataset generation and definition of Periodontitis Risk Score (PRS)

To train our neural network model, we required a substantial dataset. This was achieved by analysing real-world 16S sequencing profiling data and generating a synthetic dataset. The dataset construction process is detailed as follows.

Initially, the 16S sequencing profiles in CON and PD groups were represented as OC [Oral Microbe Control] ([o, c]) and OP [Oral Microbe Periodontitis] ([o, p]), respectively. We began with 454 oral microbes, where ‘r’ is the identification number for each oral microbe and ‘c’ and ‘p’ identify participants in the CON and PD groups, respectively.

After filtering and selection, the oral microbes in CON and PD groups were represented as SC [selected oral microbe, control] ([s, c]) and SP [selected oral microbe, periodontitis] ([s, p]). Here, ‘s’ denotes the number of oral microbe postfiltration as determined by the parameters of the preprocessing algorithm.

Using these filtered oral microbes, we constructed the training dataset for the proposed PSPNet model. The training dataset comprised *x*_train_ and *y*_train_, while the validation dataset comprised *x*_test_ and *y*_test_. In this context, _train_ and *x*_test_ represent the oral microbe abundance matrices, and *y*_train_ and *y*_test_ are the corresponding probability vectors contributing to PD risk. The mean value of the PD probability vector was denoted as the PRS. Our dataset included: (1) actual oral microbe abundance from 16S rRNA sequencing data of dental plaque, and (2) values generated randomly using a Gaussian function. The expression quantities in *x*_train_ and *x*_test_ adhered to practical sequencing range distributions. The dataset generation using the Gaussian function is outlined in the following equation:

(1) $$\left\{ \begin{array}{c} x\_train[i]=rands\{Max[Max(SP[i]),Max(SC[i])],Min[Min(SP[i]),Min(SC[i])],M\}\\ x\_test[i]=rands\{Max[Max(SP[i]),Max(SC[i])],Min[Min(SP[i]),Min(SC[i])],Q\} \end{array}\right.\;i\in \left[1,2,3,\cdots ,N\right]$$

Here, rands() denotes the Gaussian random function, Max() and Min() are the maximum and minimum value functions, and M and Q are the numbers of samples generated. We calculated each oral microbe's contribution to the occurrence of CON or PD based on the abundance and clinical diagnosis (prior knowledge) of SC [s, c] and SP [s, p]. For instance, if the abundance of a microbe was significantly higher in the PD group than in the Con group and reached the maximum among all detected oral microbes, it was assigned a contribution value of ‘1’ to PD, and vice versa.

The vector set of oral microbe contributions (*y*_train_ and *y*_test_) was computed from *x*_train_ and *x*_test_, and clinical diagnosis (prior knowledge). This process is described by the following equations:

(2) $$\left\{ \begin{array}{c} y\_train\left[i\right]=\frac{x\_train[i]-Min(x\_train[i])}{Max(x\_Train[i])-Min(x\_train[i])},\text{if}\;avg\left(SP\left[i\right]\right)>avg\left(SC\left[i\right]\right)\\ y\_train\left[i\right]=\frac{Max(x\_train[i])-x\_train[i]}{Max(x\_train[i])-Min(x\_train[i])},\text{if}\;avg\left(SP\left[i\right]\right)<avg\left(SC\left[i\right]\right) \end{array}\right.,i\in \left[1,2,3,\cdots N\right]$$

(3) $$\left\{ \begin{array}{c} y\_test\left[i\right]=\frac{x\_test[i]-Min(x\_test[i])}{Max(x\_test[i])-Min(x\_test[i])},if\;avg\left(SP\left[i\right]\right)>avg\left(SC\left[i\right]\right)\\ y\_test\left[i\right]=\frac{Max(x\_test[i])-x\_test[i]}{Max(x\_test[i])-Min(x\_test[i])},if\;avg\left(SP\left[i\right]\right)<avg\left(SC\left[i\right]\right) \end{array}\right.,i\in \left[1,2,3,\cdots N\right]$$

where avg() is the average value function, and *N* = *s*.

Ultimately, we obtained *x*_train_ and *y*_train_ with dimensions [M, N] and *x*_test_ and *y*_test_ with dimensions [Q, N]. These values were reshaped to [1, N, M] and [1, N, Q], respectively, through dimension transformation. Here, *N* = *s, M* = 8000, and *Q* = 500, indicating that there were 8000 training sample vectors in 1 × *N* and 500 validation sample vectors in 1 × *N*. The PRS was computed as the average of *y*_test_. Table provides details of the dataset.

**Table:** Dataset for PSPNet model training and validation.

Dataset	Dimension	Transformed dimension	Note
x_train	[M, N] *N* = *s*	[1, N, M] M = 8000	Training dataset (oral microbes abundance matrices)
y_train	[M, N] *N* = *s*	[1, N, M] M = 8000	Training dataset (periodontitis risk score)
x_test	[Q, N] *N* = *s*	[1, N, Q] Q = 500	Validation dataset (oral microbes abundance matrices)
y_test	[Q, N] *N* = *s*	[1, N, Q] Q = 500	Validation dataset (periodontitis risk score)
X_SampleCon	[Pc, N] *N* = *s*	[1, N, Pc] Pc = 90	prediction dataset (oral microbes abundance matrices)
X_SamplePD	[Pp, N] *N* = *s*	[1, N, Pp] Pp = 514	prediction dataset (oral microbes abundance matrices)

Note: *s* is selected oral microbes abundance matrices that have significant contribution for PD risk whose value is *s* = 27.

### PSPNet construction

#### Model architecture

The PSPNet was designed to predict the PRS using microbial profiling data collected from CON and PD groups (Figure 1C). The model comprises three primary modules: the convolutional neural network (CNN) module, the Pyramid Pooling module, and the Output module (Figure 1D).

#### CNN module

The CNN module serves as the feature extraction layer. It extracts feature maps from the oral microbe abundance matrices, capturing essential low-level semantic information for subsequent layers. The configuration of this module includes (1) Convolutional layers with a kernel size of 3 × 3, 128 channels, and a stride of 1; (2) activation function: ReLU (Rectified Linear Unit).

#### Pyramid pooling module

This module employs a pyramid structure to capture multiscale global contextual features from each subregion. Intermediate feature maps are further processed through max-pooling layers to produce refined feature maps at different scales. The convolutional layers extract semantic information from low to high levels. Convolutional kernel size is 3 × 3, with 128 channels and a stride of 5, ReLU is adopted as activation function.

#### Output module

The Output module concatenates local pointwise features with the learned multiscale contextual features, resulting in more accurate predictions. The final PD probability vector is generated by a dense layer, revealing the contributions of significant microbial indicators to PD risk. The configuration of this module includes (1) Convolutional layers with a kernel size of 3 × 3, 128 channels, and a stride of 1. (2) A dense layer with a kernel size of 1 × 1 and 128 channels. (3) Activation function: ReLU.

### Preprocessing and data filtering

Before inputting the data into PSPNet, the microbial profiling data undergo preprocessing to filter out oral microbes with significant features. This step ensures that only the most relevant oral microbes are used as input, with the predicted PD probability, namely PRS as the output.

### Overfitting prevention

To mitigate overfitting during model training, Batch Normalization layers are inserted before each convolutional layer to normalize input features. Additionally, a dropout operator with a parameter set to 0.2 is added after the convolutional layers to prevent overfitting by randomly deactivating a fraction of the neurons during training.

### Training configuration

The training of PSPNet is configured with the following hyperparameters. Optimizer: Adam; learning rate: 0.0005; loss function: mean absolute error (MAE); metrics: accuracy; batch size: 16; epochs: 500; validation split: 0.05.

The MAE loss function is defined as:

$$MAE\left(y_{i},{\hat{y}}_{i}\right)=\frac{1}{N}\sum_{i=1}^{N}\left|y_{i}-{\hat{y}}_{i}\right|$$

where *y_i_* is the ground truth of PD probability, *y_i_*^ is the predicted PD probability, and *N* is the number of samples.

### Computational efficiency

PSPNet offers an effective global contextual prior for single oral microbe-level scene parsing, significantly enhancing its ability to handle complex and large datasets efficiently. The pyramid pooling module collects multilevel information more representatively than global pooling, without substantially increasing computational cost compared to the original dilated Fully Convolutional Network. Both the global pyramid pooling module and the local Fully Convolutional Network features are optimized simultaneously in end-to-end learning, ensuring efficient processing. This efficient computational design enables PSPNet to rapidly process microbial profiling data, making it suitable for large-scale applications such as population screenings.

## Hardware configuration

The PSPNet model was trained and validated on a computer with the following specifications. CPU: Intel Core i5 13600K 3.5GHz 14C/20T; RAM: DDR4 3000MHz 32GB; GPU: Nvidia RTX2080Ti 11GB; Storage: SSD M.2 3600Mb/s 1TB. This hardware configuration ensured efficient handling of the computational demands during the PSPNet training process.

## Results

### Filtration of PD indicators

The dataset used in this study consisted of dental plaque samples from 90 CON and 514 patients with PD. Initially, microbial profiling techniques detected 454 oral microbial taxa across these samples. To identify the most relevant microbial indicators of PD, the data underwent rigorous filtration and preprocessing. Detailed preprocessing parameters are outlined in Table. Several tests were conducted to optimize the algorithm parameters, allowing us to identify microbial taxa that exhibited significant differences between the CON and PD groups. This process resulted in the selection of 27 oral microbes as key indicators of PD risk, based on their substantial differences in abundance between the two groups (see Supplemental Table 2).

### Clinical Ground Truth and Calculated Ground Truth

**Fig. 2:**
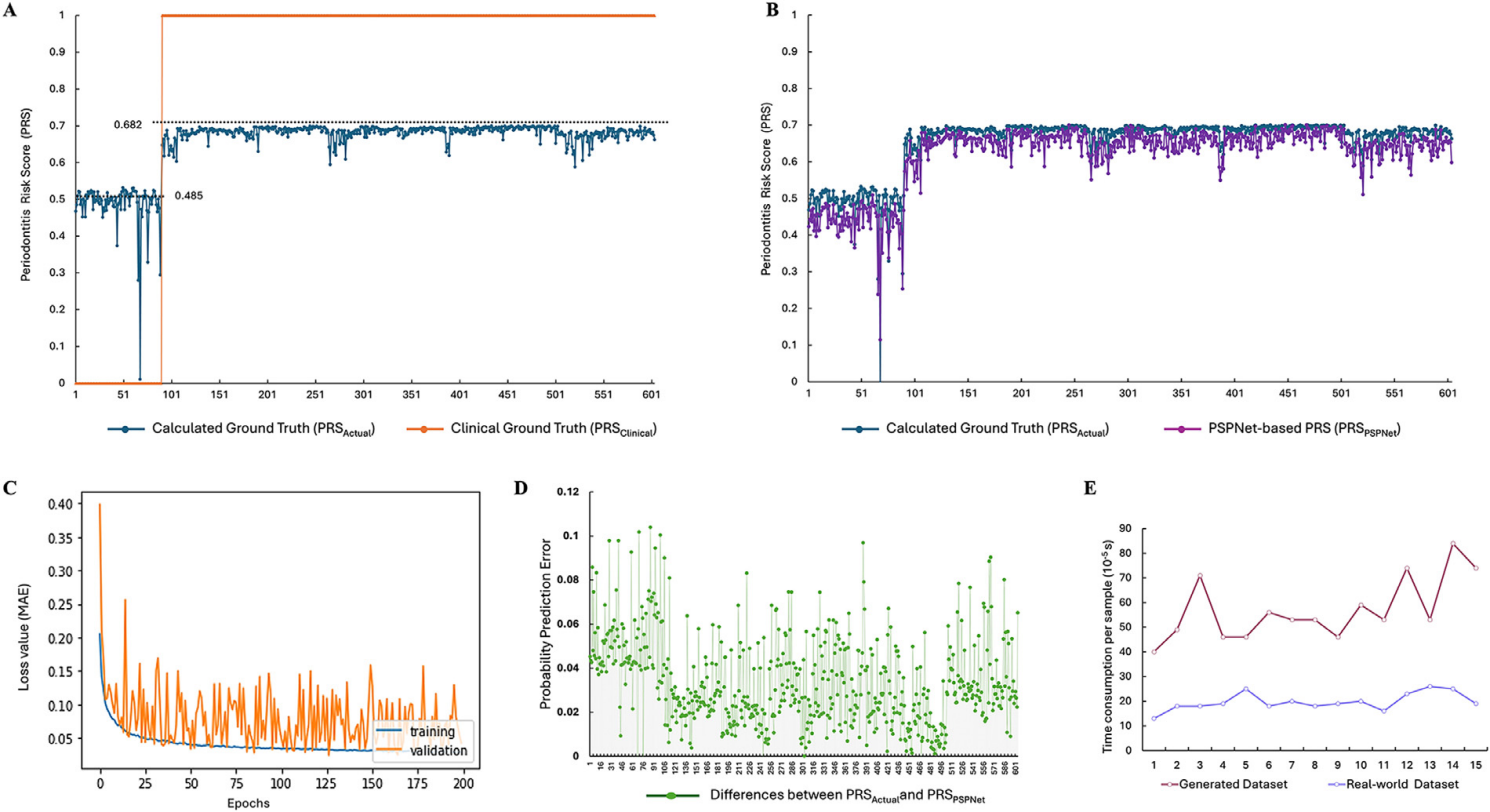
Characteristics of PSPNet in reporting periodontitis risk score. (A) The comparison between Clinical Ground Truth, namely PRS_Clinical_ (yellow dot line) and Calculated Ground Truth, namely PRS_Actual_ (blue dot line); (B) the comparison between the PRS_Actual_ (blue dot line) and PRS _PSPNet_ (purple dot line); (C) MAE between the PRS_Actual_ and the PRS _PSPNet_ after training and validation; (D) prediction error of PRS _PSPNet_; (E) time efficiency of PSPNet-based PRS prediction.

Clinical diagnosis outcomes for the enrolled subjects were categorized as either CON or PD. Thus, the Clinical Ground Truth (Clinical PRS, PRS_Clincial_) for subjects in the PD group was defined as ‘1’, while for those in the CON group, it was defined as ‘0’. As shown in Figure 2A, the orange dotted line represents the clinical PRS values, with distinct scores of 1 for the PD group and 0 for the CON group. We processed the microbial profiling data using Equations (2) and (3) to derive their Calculated Ground Truth (Calculated PRS, namely PRS_Actual_). Our results indicated that the PRS_Actual_ for the CON group ranged from 0.011 to 0.524, with an average of 0.485. In contrast, the PRS_Actual_ for the PD group ranged from 0.589 to 0.700, with an average of 0.682. The blue dots illustrate the range of PRS_Actual_ values for the CON group, while the red dots represent the calculated PRS values for the PD group. These results clearly differentiate between PD and CON subjects, confirming the reliability of PRS_Actual_ in distinguishing healthy individuals from those with PD.

### PSPNet-based PRS Verification

To validate the model's accuracy, real-world microbial profiling data were used as the test input set *x*_test for the PSPNet model to generate the PSPNet-based PRS (PRS_PSPnet_). The predicted PRS_PSPnet_ closely aligned with PRS_Actual_, demonstrating the model's precise fitting ability (Figure 2B). Through training and validation, the PSPNet model achieved optimized performance, reducing the MAE between PRS_PSPnet_ and PRS_Actual_ to 0.027 (Figure 2C). This low MAE reflects the model's high accuracy in predicting PD risk, underscoring its robustness and reliability in a clinical setting.

### Prediction parameters of PSPNet

The error amplitude of the PRS_PSPnet_ is shown in Figure 2D. The maximum absolute error, peak-to-valley error, and MAE were 0.125, 0.228, and 0.033, respectively. These values demonstrate that the PSPNet model maintains a low prediction error, highlighting its strong data-fitting capabilities.

Additionally, we evaluated the processing efficiency for large datasets in population screenings, using prediction time as a benchmark. The time required to generate a PRS value was recorded for each set of 16S rRNA profiles fed into the trained PSPNet model. As shown in Figure 2E, 15 consecutive experiments demonstrated that the average time to generate a PRS was 10^–5^ seconds per sample. This result indicates the model's high efficiency and suitability for large-scale applications.

## Discussion

This study presents a PSPNet model integrated with microbial profiling of dental plaque. The PRS_Actual_ range ranged from 0.011 to 0.524 (mean 0.485) for CON and from 0.589 to 0.700 (mean 0.682) for PD patients, successfully distinguishing between the groups. The MAE between PRS_PSPnet_ and PRS_Actual_ was 0.027, with an average computation time per sample of 10^–5^ seconds. This model provides a reliable, efficient, economical, and noninvasive tool for identifying individuals at high risk of PD.

Typically, PD is detected only after significant and irreversible tissue damage has occurred.^5^^,^^6^ Early detection allows for preventive strategies, including improved oral hygiene, regular dental check-ups, lifestyle changes, and specialized periodontal care,^10^ highlighting the importance of early diagnosis. While recent biomarker-based approaches show promise, they also have limitations. Salivary biomarkers like interleukin-1β, matrix metalloproteinase-8, and ICTP, as well as gingival crevicular fluid biomarkers such as MIP-1α and matrix metalloproteinase-9, have demonstrated differences between healthy and PD-affected individuals. However, these markers can be influenced by factors such as the oral environment, diet, and other diseases, affecting their stability and reliability. This highlights the need for more reliable methods that are less susceptible to external influences.

The role of oral microbiota in PD is well-established.^8^^,^^19^ An individual's plaque microbiota remains relatively stable over time, making it a reliable marker for oral health and supporting the feasibility of predicting PD risk based on oral microbiome composition.^8^ However, traditional data processing methods focus on the abundance of individual bacterial species as biomarkers, limiting their ability to handle large, complex sequencing datasets. These methods fail to capture the overall patterns of the oral microbiome, making it difficult to fully leverage the comprehensive data provided by microbial profiling for disease prediction based on the entire microbial community.

Using deep learning for microbial research offers significant advantages over traditional methods, which often struggle with the complexity and high dimensionality of microbiome data.^13^, ^14^, ^15^, ^16^ These models excel at handling complex datasets and capturing characteristics from a holistic perspective. By combining real-world microbial profiling with deep learning techniques, we effectively captured the complex microbial profiles associated with PD and used these profiles to train the PSPNet model. Our model clearly distinguished between PD and CON groups, with clinical PRS values of 1 for the PD group and 0 for the CON group, and PRS_Actual_ values ranging from 0.589 to 0.700 for PD and 0.011 to 0.524 for CON. This significant difference underscores the model's reliability in identifying PD risk. The PSPNet model achieved an MAE of 0.027 in probability prediction, highlighting the model's robustness and its potential clinical utility for early identification of individuals at high risk of developing PD. Additionally, the model's rapid processing time, averaging 10^−5^ seconds per sample, makes it feasible for large-scale population screenings. The PSPNet model facilitates early identification of high-risk individuals, which could significantly lessen the disease's related oral and systemic negative impacts and substantially reduce the economic burden for individuals and society, potentially preventing subsequent oral diseases and easing the financial strain on healthcare insurance systems that cover PD-related treatment costs.

Despite the promising results, this study has several limitations. Deep learning models like PSPNet are prone to overfitting, particularly with high-dimensional data such as microbial profiles, which may lead to poor performance on unseen data. Additionally, the relatively small sample size of real-world microbial profiling data may limit the model's ability to capture the full variability of human oral microbiomes. Racial and ethnic differences, along with hereditary factors, individual variations, preanalytical conditions, and technical issues such as background noise and batch effects, can also influence microbial abundance, affecting reproducibility and specificity. Future research should validate the model on larger, more diverse populations, accounting for racial and ethnic variability, and standardize sequencing and data processing methods to improve consistency and reliability.

Overall, our PSPNet model shows great potential as a noninvasive, efficient tool for the early detection of PD. Future studies addressing the model's current constraints will be crucial to refining its clinical applicability and expanding its use across diverse populations, ultimately contributing to better preventative care and reduced healthcare costs.

## Author contributions

Zhuo Zhao: Contributed to conception, design, and analysis; draft the manuscript and critically revised the manuscript; give final approval and agree to be accountable for all aspects of work ensuring integrity and accuracy. Xiaoxu Liu and Mengting Li: Contributed to conception, design, and acquisition; draft the manuscript; give final approval and agree to be accountable for all aspects of work ensuring integrity and accuracy. Jinjun Liu: Contributed to conception, design, and interpretation; draft the manuscript and critically revised the manuscript; give final approval and agree to be accountable for all aspects of work ensuring integrity and accuracy. Zheng Wang: Contributed to conception, design, analysis, and interpretation; draft the manuscript and critically revised the manuscript; give final approval and agree to be accountable for all aspects of work ensuring integrity and accuracy. All authors gave their final approval and agreed to be accountable for all aspects of the work.

## Conflict of interest

The authors declare that they have no known competing financial interests or personal relationships that could have appeared to influence the work reported in this article.

## Appendix Supplementary materials

Image, application 1

Image, application 2

## Footnotes

Supplementary material associated with this article can be found in the online version at doi:10.1016/j.identj.2024.10.019.
