# Supplementary material for: Oral Microbe Community and Pyramid Scene Parsing Network-based Periodontitis Risk Prediction
Source: Int Dent J. 2024 Nov 28;75(2):700–6. doi: 10.1016/j.identj.2024.10.019 (PMC11976633; doi:10.1016/j.identj.2024.10.019)
Supplement: Supplementary file 1 [file mmc1.doc]

STROBE Statement—checklist of items that should be included in reports of observational studies

|  | Item No | Recommendation |
| --- | --- | --- |
| **Title and abstract** | 1 | (*a*) Indicate the study’s design with a commonly used term in the title or the abstract  **Response**: The title indicates the study design as an observational study using a deep learning model. |
|  |  | (*b*) Provide in the abstract an informative and balanced summary of what was done and what was found  **Response**: The abstract provides a summary of the aim, methods, results, and conclusion of the study. |
| Introduction | | |
| Background/rationale | 2 | Explain the scientific background and rationale for the investigation being reported  **Response**: Page 1, Paragraph 1: "Periodontitis (PD) is a prevalent chronic inflammatory disease that affects the gums and supporting structures of teeth..." |
| Objectives | 3 | State specific objectives, including any prespecified hypotheses  **Response**: Page 1, Paragraph 2: "This study aims to integrate the Pyramid Scene Parsing Network (PSPNet)..." |
| Methods | | |
| Study design | 4 | Present key elements of study design early in the paper  **Response**: Page 2, Paragraph 1: Describes it as an observational study using existing data. |
| Setting | 5 | Describe the setting, locations, and relevant dates, including periods of recruitment, exposure, follow-up, and data collection  **Response**: Page 2, Paragraph 1: Describes the data source (GEO database) and the period during which the data were collected. |
| Participants | 6 | (*a*) *Cohort study*—Give the eligibility criteria, and the sources and methods of selection of participants. Describe methods of follow-up  *Case-control study*—Give the eligibility criteria, and the sources and methods of case ascertainment and control selection. Give the rationale for the choice of cases and controls  *Cross-sectional study*—Give the eligibility criteria, and the sources and methods of selection of participants  **Response**: Page 2, Paragraph 1: Explains the criteria for selecting the healthy controls and PD patients from the GEO database. |
|  |  | (*b*) *Cohort study*—For matched studies, give matching criteria and number of exposed and unexposed  *Case-control study*—For matched studies, give matching criteria and the number of controls per case |
| Variables | 7 | Clearly define all outcomes, exposures, predictors, potential confounders, and effect modifiers. Give diagnostic criteria, if applicable  **Response**: Page 2, Paragraph 2: Defines the PRS, the indicators used, and the PSPNet model. |
| Data sources/ measurement | 8* | For each variable of interest, give sources of data and details of methods of assessment (measurement). Describe comparability of assessment methods if there is more than one group  **Response**: Page 2, Paragraph 1: Source of 16S rRNA sequencing data. |
| Bias | 9 | Describe any efforts to address potential sources of bias  **Response**: Page 2, Paragraph 2: Efforts to address potential sources of bias. |
| Study size | 10 | Explain how the study size was arrived at  **Response**: Page 2, Paragraph 1: Number of controls and PD patients used in the study. |
| Quantitative variables | 11 | Explain how quantitative variables were handled in the analyses. If applicable, describe which groupings were chosen and why  **Response**: Page 2, Paragraph 3: Handling of quantitative variables. |
| Statistical methods | 12 | (*a*) Describe all statistical methods, including those used to control for confounding  **Response**: Page 2, Paragraph 4: Statistical methods used, control for confounding. |
|  |  | (*b*) Describe any methods used to examine subgroups and interactions  **Response**: Not applicable. |
|  |  | (*c*) Explain how missing data were addressed  **Response**: Not applicable. |
|  |  | (*d*) *Cohort study*—If applicable, explain how loss to follow-up was addressed  *Case-control study*—If applicable, explain how matching of cases and controls was addressed  *Cross-sectional study*—If applicable, describe analytical methods taking account of sampling strategy  **Response**: Not applicable. |
|  |  | (*e*) Describe any sensitivity analyses  **Response**: Not applicable. |

Continued on next page

| Results | | |
| --- | --- | --- |
| Participants | 13* | (a) Report numbers of individuals at each stage of study—eg numbers potentially eligible, examined for eligibility, confirmed eligible, included in the study, completing follow-up, and analysed  **Response**: Page 3, Paragraph 1: Numbers at each stage of the study. |
|  |  | (b) Give reasons for non-participation at each stage  **Response**: Not applicable. |
|  |  | (c) Consider use of a flow diagram  **Response**: Not applicable. |
| Descriptive data | 14* | (a) Give characteristics of study participants (eg demographic, clinical, social) and information on exposures and potential confounders  **Response**: Page 3, Paragraph 1: Characteristics of study participants. |
|  |  | (b) Indicate number of participants with missing data for each variable of interest  **Response**: Not applicable. |
|  |  | (c) *Cohort study*—Summarise follow-up time (eg, average and total amount)  **Response**: Not applicable. |
| Outcome data | 15* | *Cohort study*—Report numbers of outcome events or summary measures over time |
|  |  | *Case-control study—*Report numbers in each exposure category, or summary measures of exposure |
|  |  | *Cross-sectional study—*Report numbers of outcome events or summary measures  **Response**: Page 3, Paragraph 1: Report of outcome events. |
| Main results | 16 | (*a*) Give unadjusted estimates and, if applicable, confounder-adjusted estimates and their precision (eg, 95% confidence interval). Make clear which confounders were adjusted for and why they were included  **Response**: Page 3, Paragraph 2: Unadjusted and adjusted estimates. |
|  |  | (*b*) Report category boundaries when continuous variables were categorized  **Response**: Not applicable. |
|  |  | (*c*) If relevant, consider translating estimates of relative risk into absolute risk for a meaningful time period  **Response**: Not applicable. |
| Other analyses | 17 | Report other analyses done—eg analyses of subgroups and interactions, and sensitivity analyses  **Response**: Page 3, Paragraph 3: Report of subgroup analyses. |
| Discussion | | |
| Key results | 18 | Summarise key results with reference to study objectives  **Response**: Page 4, Paragraph 1: Summarizes key results. |
| Limitations | 19 | Discuss limitations of the study, taking into account sources of potential bias or imprecision. Discuss both direction and magnitude of any potential bias  **Response**: Page 4, Paragraph 2: Discusses study limitations. |
| Interpretation | 20 | Give a cautious overall interpretation of results considering objectives, limitations, multiplicity of analyses, results from similar studies, and other relevant evidence  **Response**: Page 4, Paragraph 3: Overall interpretation of results. |
| Generalisability | 21 | Discuss the generalisability (external validity) of the study results  **Response**: Page 4, Paragraph 4: Discusses generalisability of results. |
| Other information | | |
| Funding | 22 | Give the source of funding and the role of the funders for the present study and, if applicable, for the original study on which the present article is based  **Response**: Page 5, Paragraph 1: Source of funding and role of funders. |

*Give information separately for cases and controls in case-control studies and, if applicable, for exposed and unexposed groups in cohort and cross-sectional studies.

**Note:** An Explanation and Elaboration article discusses each checklist item and gives methodological background and published examples of transparent reporting. The STROBE checklist is best used in conjunction with this article (freely available on the Web sites of PLoS Medicine at http://www.plosmedicine.org/, Annals of Internal Medicine at http://www.annals.org/, and Epidemiology at http://www.epidem.com/). Information on the STROBE Initiative is available at www.strobe-statement.org.
