# Supplementary material for: Oral Microbe Community and Pyramid Scene Parsing Network-based Periodontitis Risk Prediction
Source: Int Dent J. 2024 Nov 28;75(2):700–6. doi: 10.1016/j.identj.2024.10.019 (PMC11976633; doi:10.1016/j.identj.2024.10.019)
Supplement: Supplementary file 2 [file mmc2.docx]

| **Table 2 Selected indicators for periodontitis prediction** | | | | | | | | |
| --- | --- | --- | --- | --- | --- | --- | --- | --- |
| ***Oral Microbe*** | **Taxonomic Rank** | **Gram Staining** | **Role in Oral Health** | **Associated Diseases** | | **Habitat** | **Prevalence in Periodontitis** | **Virulence Factors** |
| *Achromobacter xylosoxidans_AC18* | Species | Negative | Pathogenic | Respiratory infections, periodontitis | | Oral cavity | Medium | Biofilm formation |
| *Bacteroides heparinolyticus I_N91* | Species | Negative | Pathogenic | Periodontitis | | Subgingival plaque | High | Proteolytic enzymes |
| *Bifidobacterium sp strain A32ED I_X51* | Species | Positive | Pathogenic | Gastrointestinal disorders | | Oral and gut | Low | Acid resistance |
| *Burkholderia cepacia_AC05* | Species | Negative | Pathogenic | Respiratory infections | | Oral cavity | Medium | Antibiotic resistance |
| *Burkholderia Cluster I_AB64* | Species | Negative | Pathogenic | Cystic fibrosis, periodontitis | | Oral cavity | Medium | Biofilm formation |
| *Burkholderia Cluster II_AB65* | Species | Negative | Pathogenic | Cystic fibrosis, periodontitis | | Oral cavity | Medium | Biofilm formation |
| *Enterococcus faecalis I_N28* | Species | Positive | Pathogenic | Endocarditis, periodontitis | | Oral cavity | High | Biofilm formation, exotoxins |
| *Enterococcus faecalis II_AB12* | Species | Positive | Pathogenic | Endocarditis, periodontitis | | Oral cavity | High | Biofilm formation, exotoxins |
| *Enterococcus italicus_saccharominimus II_AB45* | Species | Positive | Pathogenic | Periodontitis | | Oral cavity | Medium | Biofilm formation |
| *Eubacterium minutum_AC65* | Species | Positive | Pathogenic | Periodontitis, systemic infections | | Oral cavity | Medium | Butyrate production |
| *Neisseria pharyngis I_K71* | Species | Negative | Commensal | Rarely pathogenic | | Oral cavity | Low | - |
| *OP11 ANUG X112 I_AC72* | Species | Negative | Pathogenic | Acute necrotizing ulcerative gingivitis | | Oral cavity | High | Toxins, enzymes |
| *Porphyromonas catoniae_EP003 II_AA91* | Species | Negative | Pathogenic | Periodontitis | | Subgingival plaque | High | Proteases, fimbriae |
| *Porphyromonas Cluster I_AA46* | Species | Negative | Pathogenic | Periodontitis | | Subgingival plaque | High | Proteases, fimbriae |
| *Prevotella multisaccharivorax I_AC58* | Species | Negative | Pathogenic | Periodontitis | | Subgingival plaque | High | Proteases |
| *Prevotella sp AH125 I_N86* | Species | Negative | Pathogenic | Periodontitis | | Subgingival plaque | High | Proteases |
| *Prevotella sp AH125 II_P68* | Species | Negative | Pathogenic | Periodontitis | | Subgingival plaque | High | Proteases |
| *Prevotella sp BI027 II_AA51* | Species | Negative | Pathogenic | Periodontitis | | Subgingival plaque | High | Proteases |
| *Prevotella sp DO045 I_Q97* | Species | Negative | Pathogenic | | Periodontitis | Subgingival plaque | High | Proteases |
| *Ralstonia Cluster I_AC20* | Species | Negative | Pathogenic | | Respiratory infections | Oral cavity | Medium | Biofilm formation |
| *Selenomonas sp DM071 I_AC62* | Species | Negative | Pathogenic | | Periodontitis | Subgingival plaque | High | Flagella |
| *Selenomonas sp IQ048 II_AB57* | Species | Negative | Pathogenic | | Periodontitis | Subgingival plaque | High | Flagella |
| *Streptococcus australis_Dialister invisus I_U50* | Species | Positive | Pathogenic | | Dental caries, Periodontitis | Oral cavity | Medium | Acid production |
| *Streptococcus cristatus_infantis I_X08* | Species | Positive | Commensal | | Rarely pathogenic | Oral cavity | Low | - |
| *Streptococcus downei II_AB80* | Species | Positive | Pathogenic | | Dental caries, periodontitis | Oral cavity | Medium | Acid production |
| *Synergistes sp W028 I_O36* | Species | Negative | Pathogenic | | Periodontitis | Subgingival plaque | Medium | Hydrogen sulfide production |
| *Veillonella Cluster III_W87* | Species | Negative | Commensal | | Rarely pathogenic | Oral cavity | Low | - |
